# Supplementary material for: Resolving the full spectrum of human genome variation using Linked-Reads
Source: Genome Res. 2019 Apr;29(4):635–45. doi: 10.1101/gr.234443.118 (PMC6442396; doi:10.1101/gr.234443.118)
Supplement: Supplemental Material [file supp_gr.234443.118_Supplemental_longranger-2.2.zip › longranger-2.2.tar.bz2!/lr2.2-public/lib/stan/cmdstan/stan/doc/api/html/beta_8hpp_source.html]

Stan: src/stan/prob/distributions/univariate/continuous/beta.hpp Source File


|  |  |
| --- | --- |
|  | Stan  2.5.0  probability, sampling & optimization |


- Main Page
- Related Pages
- Namespaces
- Classes
- Files

- File List
- File Members

All Classes Namespaces Files Functions Variables Typedefs Enumerations Enumerator Friends Macros Pages

- src
- stan
- prob
- distributions
- univariate
- continuous

beta.hpp

Go to the documentation of this file.

1 #ifndef STAN\_\_PROB\_\_DISTRIBUTIONS\_\_UNIVARIATE\_\_CONTINUOUS\_\_BETA\_HPP

2 #define STAN\_\_PROB\_\_DISTRIBUTIONS\_\_UNIVARIATE\_\_CONTINUOUS\_\_BETA\_HPP

3

4 #include <boost/math/special\_functions/gamma.hpp>

5 #include <boost/random/gamma\_distribution.hpp>

6 #include <boost/random/variate\_generator.hpp>

7

8 #include <stan/agrad/partials\_vari.hpp>

9 #include <stan/math/error\_handling.hpp>

10 #include <stan/math/functions/log1m.hpp>

11 #include <stan/math/functions/multiply\_log.hpp>

12 #include <stan/math/functions/value\_of.hpp>

13 #include <stan/meta/traits.hpp>

14 #include <stan/prob/constants.hpp>

15 #include <stan/prob/traits.hpp>

16 #include <stan/prob/internal\_math.hpp>

17

18 namespace stan {

19

20  namespace prob {

21

43  template <bool propto,

44  typename T\_y, typename T\_scale\_succ, typename T\_scale\_fail>

45  typename return\_type<T\_y,T\_scale\_succ,T\_scale\_fail>::type

46  beta\_log(const T\_y& y,

47  const T\_scale\_succ& alpha, const T\_scale\_fail& beta) {

48  static const char\* function = "stan::prob::beta\_log(%1%)";

49

50  using boost::math::digamma;

51  using boost::math::lgamma;

52  using stan::is\_constant\_struct;

53  using stan::is\_vector;

54  using stan::math::check\_positive\_finite;

55  using stan::math::check\_not\_nan;

56  using stan::math::check\_consistent\_sizes;

57  using stan::prob::include\_summand;

58  using stan::math::log1m;

59  using stan::math::multiply\_log;

60  using stan::math::value\_of;

61  using stan::math::check\_nonnegative;

62  using stan::math::check\_less\_or\_equal;

63

64  // check if any vectors are zero length

65  if (!(stan::length(y)

66  && stan::length(alpha)

67  && stan::length(beta)))

68  return 0.0;

69

70  // set up return value accumulator

71  double logp(0.0);

72

73  // validate args (here done over var, which should be OK)

74  check\_positive\_finite(function, alpha, "First shape parameter", &logp);

75  check\_positive\_finite(function, beta, "Second shape parameter", &logp);

76  check\_not\_nan(function, y, "Random variable", &logp);

77  check\_consistent\_sizes(function,

78  y,alpha,beta,

79  "Random variable","First shape parameter",

80  "Second shape parameter",

81  &logp);

82  check\_nonnegative(function, y, "Random variable", &logp);

83  check\_less\_or\_equal(function, y, 1,"Random variable", &logp);

84

85  // check if no variables are involved and prop-to

86  if (!include\_summand<propto,T\_y,T\_scale\_succ,T\_scale\_fail>::value)

87  return 0.0;

88

89  VectorView<const T\_y> y\_vec(y);

90  VectorView<const T\_scale\_succ> alpha\_vec(alpha);

91  VectorView<const T\_scale\_fail> beta\_vec(beta);

92  size\_t N = max\_size(y, alpha, beta);

93

94  for (size\_t n = 0; n < N; n++) {

95  const double y\_dbl = value\_of(y\_vec[n]);

96  if (y\_dbl < 0 || y\_dbl > 1)

97  return LOG\_ZERO;

98  }

99

100  // set up template expressions wrapping scalars into vector views

101  agrad::OperandsAndPartials<T\_y, T\_scale\_succ, T\_scale\_fail>

102  operands\_and\_partials(y, alpha, beta);

103

104  DoubleVectorView<include\_summand<propto,T\_y,T\_scale\_succ>::value,

105  is\_vector<T\_y>::value> log\_y(length(y));

106  DoubleVectorView<include\_summand<propto,T\_y,T\_scale\_fail>::value,

107  is\_vector<T\_y>::value> log1m\_y(length(y));

108

109  for (size\_t n = 0; n < length(y); n++) {

110  if (include\_summand<propto,T\_y,T\_scale\_succ>::value)

111  log\_y[n] = log(value\_of(y\_vec[n]));

112  if (include\_summand<propto,T\_y,T\_scale\_fail>::value)

113  log1m\_y[n] = log1m(value\_of(y\_vec[n]));

114  }

115

116  DoubleVectorView<include\_summand<propto,T\_scale\_succ>::value,

117  is\_vector<T\_scale\_succ>::value> lgamma\_alpha(length(alpha));

118  DoubleVectorView<!is\_constant\_struct<T\_scale\_succ>::value,

119  is\_vector<T\_scale\_succ>::value> digamma\_alpha(length(alpha));

120  for (size\_t n = 0; n < length(alpha); n++) {

121  if (include\_summand<propto,T\_scale\_succ>::value)

122  lgamma\_alpha[n] = lgamma(value\_of(alpha\_vec[n]));

123  if (!is\_constant\_struct<T\_scale\_succ>::value)

124  digamma\_alpha[n] = digamma(value\_of(alpha\_vec[n]));

125  }

126

127  DoubleVectorView<include\_summand<propto,T\_scale\_fail>::value,

128  is\_vector<T\_scale\_fail>::value> lgamma\_beta(length(beta));

129  DoubleVectorView<!is\_constant\_struct<T\_scale\_fail>::value,

130  is\_vector<T\_scale\_fail>::value> digamma\_beta(length(beta));

131

132  for (size\_t n = 0; n < length(beta); n++) {

133  if (include\_summand<propto,T\_scale\_fail>::value)

134  lgamma\_beta[n] = lgamma(value\_of(beta\_vec[n]));

135  if (!is\_constant\_struct<T\_scale\_fail>::value)

136  digamma\_beta[n] = digamma(value\_of(beta\_vec[n]));

137  }

138

139  DoubleVectorView<include\_summand<propto,T\_scale\_succ,T\_scale\_fail>::value,

140  is\_vector<T\_scale\_succ>::value

141  || is\_vector<T\_scale\_fail>::value>

142  lgamma\_alpha\_beta(max\_size(alpha,beta));

143

144  DoubleVectorView<!is\_constant\_struct<T\_scale\_succ>::value

145  || !is\_constant\_struct<T\_scale\_fail>::value,

146  is\_vector<T\_scale\_succ>::value

147  || is\_vector<T\_scale\_fail>::value>

148  digamma\_alpha\_beta(max\_size(alpha,beta));

149

150  for (size\_t n = 0; n < max\_size(alpha,beta); n++) {

151  const double alpha\_beta = value\_of(alpha\_vec[n])

152  + value\_of(beta\_vec[n]);

153  if (include\_summand<propto,T\_scale\_succ,T\_scale\_fail>::value)

154  lgamma\_alpha\_beta[n] = lgamma(alpha\_beta);

155  if (!is\_constant\_struct<T\_scale\_succ>::value

156  || !is\_constant\_struct<T\_scale\_fail>::value)

157  digamma\_alpha\_beta[n] = digamma(alpha\_beta);

158  }

159

160  for (size\_t n = 0; n < N; n++) {

161  // pull out values of arguments

162  const double y\_dbl = value\_of(y\_vec[n]);

163  const double alpha\_dbl = value\_of(alpha\_vec[n]);

164  const double beta\_dbl = value\_of(beta\_vec[n]);

165

166  // log probability

167  if (include\_summand<propto,T\_scale\_succ,T\_scale\_fail>::value)

168  logp += lgamma\_alpha\_beta[n];

169  if (include\_summand<propto,T\_scale\_succ>::value)

170  logp -= lgamma\_alpha[n];

171  if (include\_summand<propto,T\_scale\_fail>::value)

172  logp -= lgamma\_beta[n];

173  if (include\_summand<propto,T\_y,T\_scale\_succ>::value)

174  logp += (alpha\_dbl-1.0) \* log\_y[n];

175  if (include\_summand<propto,T\_y,T\_scale\_fail>::value)

176  logp += (beta\_dbl-1.0) \* log1m\_y[n];

177

178  // gradients

179  if (!is\_constant\_struct<T\_y>::value)

180  operands\_and\_partials.d\_x1[n] += (alpha\_dbl-1)/y\_dbl

181  + (beta\_dbl-1)/(y\_dbl-1);

182  if (!is\_constant\_struct<T\_scale\_succ>::value)

183  operands\_and\_partials.d\_x2[n]

184  += log\_y[n] + digamma\_alpha\_beta[n] - digamma\_alpha[n];

185  if (!is\_constant\_struct<T\_scale\_fail>::value)

186  operands\_and\_partials.d\_x3[n]

187  += log1m\_y[n] + digamma\_alpha\_beta[n] - digamma\_beta[n];

188  }

189  return operands\_and\_partials.to\_var(logp);

190  }

191

192  template <typename T\_y, typename T\_scale\_succ, typename T\_scale\_fail>

193  inline typename return\_type<T\_y,T\_scale\_succ,T\_scale\_fail>::type

194  beta\_log(const T\_y& y, const T\_scale\_succ& alpha,

195  const T\_scale\_fail& beta) {

196  return beta\_log<false>(y,alpha,beta);

197  }

198

211  template <typename T\_y, typename T\_scale\_succ, typename T\_scale\_fail>

212  typename return\_type<T\_y,T\_scale\_succ,T\_scale\_fail>::type

213  beta\_cdf(const T\_y& y, const T\_scale\_succ& alpha,

214  const T\_scale\_fail& beta) {

215

216  // Size checks

217  if ( !( stan::length(y) && stan::length(alpha)

218  && stan::length(beta) ) )

219  return 1.0;

220

221  // Error checks

222  static const char\* function = "stan::prob::beta\_cdf(%1%)";

223

224  using stan::math::check\_positive\_finite;

225  using stan::math::check\_not\_nan;

226  using boost::math::tools::promote\_args;

227  using stan::math::check\_consistent\_sizes;

228  using stan::math::value\_of;

229  using stan::math::check\_nonnegative;

230  using stan::math::check\_less\_or\_equal;

231

232  double P(1.0);

233

234  check\_positive\_finite(function, alpha, "First shape parameter", &P);

235  check\_positive\_finite(function, beta, "Second shape parameter", &P);

236  check\_not\_nan(function, y, "Random variable", &P);

237  check\_consistent\_sizes(function, y, alpha, beta,

238  "Random variable", "Shape parameter",

239  "Scale Parameter", &P);

240  check\_nonnegative(function, y, "Random variable", &P);

241  check\_less\_or\_equal(function, y, 1,"Random variable", &P);

242

243  // Wrap arguments in vectors

244  VectorView<const T\_y> y\_vec(y);

245  VectorView<const T\_scale\_succ> alpha\_vec(alpha);

246  VectorView<const T\_scale\_fail> beta\_vec(beta);

247  size\_t N = max\_size(y, alpha, beta);

248

249  agrad::OperandsAndPartials<T\_y, T\_scale\_succ, T\_scale\_fail>

250  operands\_and\_partials(y, alpha, beta);

251

252  // Explicit return for extreme values

253  // The gradients are technically ill-defined, but treated as zero

254  for (size\_t i = 0; i < stan::length(y); i++) {

255  if (value\_of(y\_vec[i]) <= 0)

256  return operands\_and\_partials.to\_var(0.0);

257  }

258

259  // Compute CDF and its gradients

260  using boost::math::ibeta;

261  using boost::math::ibeta\_derivative;

262  using boost::math::digamma;

263

264  // Cache a few expensive function calls if alpha or beta is a parameter

265  DoubleVectorView<!is\_constant\_struct<T\_scale\_succ>::value

266  || !is\_constant\_struct<T\_scale\_fail>::value,

267  is\_vector<T\_scale\_succ>::value || is\_vector<T\_scale\_fail>::value>

268  digamma\_alpha\_vec(max\_size(alpha, beta));

269

270  DoubleVectorView<!is\_constant\_struct<T\_scale\_succ>::value

271  || !is\_constant\_struct<T\_scale\_fail>::value,

272  is\_vector<T\_scale\_succ>::value || is\_vector<T\_scale\_fail>::value>

273  digamma\_beta\_vec(max\_size(alpha, beta));

274

275  DoubleVectorView<!is\_constant\_struct<T\_scale\_succ>::value

276  || !is\_constant\_struct<T\_scale\_fail>::value,

277  is\_vector<T\_scale\_succ>::value || is\_vector<T\_scale\_fail>::value>

278  digamma\_sum\_vec(max\_size(alpha, beta));

279

280  DoubleVectorView<!is\_constant\_struct<T\_scale\_succ>::value

281  || !is\_constant\_struct<T\_scale\_fail>::value,

282  is\_vector<T\_scale\_succ>::value || is\_vector<T\_scale\_fail>::value>

283  betafunc\_vec(max\_size(alpha, beta));

284

285  if (!is\_constant\_struct<T\_scale\_succ>::value

286  || !is\_constant\_struct<T\_scale\_fail>::value) {

287

288  for (size\_t i = 0; i < N; i++) {

289

290  const double alpha\_dbl = value\_of(alpha\_vec[i]);

291  const double beta\_dbl = value\_of(beta\_vec[i]);

292

293  digamma\_alpha\_vec[i] = digamma(alpha\_dbl);

294  digamma\_beta\_vec[i] = digamma(beta\_dbl);

295  digamma\_sum\_vec[i] = digamma(alpha\_dbl + beta\_dbl);

296  betafunc\_vec[i] = boost::math::beta(alpha\_dbl, beta\_dbl);

297

298  }

299

300  }

301

302  // Compute vectorized CDF and gradient

303  for (size\_t n = 0; n < N; n++) {

304

305  // Explicit results for extreme values

306  // The gradients are technically ill-defined, but treated as zero

307  if (value\_of(y\_vec[n]) >= 1.0) continue;

308

309  // Pull out values

310  const double y\_dbl = value\_of(y\_vec[n]);

311  const double alpha\_dbl = value\_of(alpha\_vec[n]);

312  const double beta\_dbl = value\_of(beta\_vec[n]);

313

314  // Compute

315  const double Pn = ibeta(alpha\_dbl, beta\_dbl, y\_dbl);

316

317  P \*= Pn;

318

319  if (!is\_constant\_struct<T\_y>::value)

320  operands\_and\_partials.d\_x1[n] += ibeta\_derivative(alpha\_dbl, beta\_dbl,

321  y\_dbl) / Pn;

322

323  double g1 = 0;

324  double g2 = 0;

325

326  if (!is\_constant\_struct<T\_scale\_succ>::value

327  || !is\_constant\_struct<T\_scale\_fail>::value) {

328  stan::math::gradRegIncBeta(g1, g2, alpha\_dbl, beta\_dbl, y\_dbl,

329  digamma\_alpha\_vec[n],

330  digamma\_beta\_vec[n], digamma\_sum\_vec[n],

331  betafunc\_vec[n]);

332  }

333

334  if (!is\_constant\_struct<T\_scale\_succ>::value)

335  operands\_and\_partials.d\_x2[n] += g1 / Pn;

336  if (!is\_constant\_struct<T\_scale\_fail>::value)

337  operands\_and\_partials.d\_x3[n] += g2 / Pn;

338  }

339

340  if (!is\_constant\_struct<T\_y>::value) {

341  for(size\_t n = 0; n < stan::length(y); ++n)

342  operands\_and\_partials.d\_x1[n] \*= P;

343  }

344  if (!is\_constant\_struct<T\_scale\_succ>::value) {

345  for(size\_t n = 0; n < stan::length(alpha); ++n)

346  operands\_and\_partials.d\_x2[n] \*= P;

347  }

348  if (!is\_constant\_struct<T\_scale\_fail>::value) {

349  for(size\_t n = 0; n < stan::length(beta); ++n)

350  operands\_and\_partials.d\_x3[n] \*= P;

351  }

352

353  return operands\_and\_partials.to\_var(P);

354  }

355

356  template <typename T\_y, typename T\_scale\_succ, typename T\_scale\_fail>

357  typename return\_type<T\_y,T\_scale\_succ,T\_scale\_fail>::type

358  beta\_cdf\_log(const T\_y& y, const T\_scale\_succ& alpha,

359  const T\_scale\_fail& beta) {

360

361  // Size checks

362  if ( !( stan::length(y) && stan::length(alpha)

363  && stan::length(beta) ) )

364  return 0.0;

365

366  // Error checks

367  static const char\* function = "stan::prob::beta\_cdf(%1%)";

368

369  using stan::math::check\_positive\_finite;

370  using stan::math::check\_not\_nan;

371  using stan::math::check\_nonnegative;

372  using stan::math::check\_less\_or\_equal;

373  using boost::math::tools::promote\_args;

374  using stan::math::check\_consistent\_sizes;

375  using stan::math::value\_of;

376

377  double cdf\_log(0.0);

378

379  check\_positive\_finite(function, alpha, "First shape parameter", &cdf\_log);

380  check\_positive\_finite(function, beta, "Second shape parameter", &cdf\_log);

381  check\_not\_nan(function, y, "Random variable", &cdf\_log);

382  check\_nonnegative(function, y, "Random variable", &cdf\_log);

383  check\_less\_or\_equal(function, y, 1,"Random variable", &cdf\_log);

384  check\_consistent\_sizes(function, y, alpha, beta,

385  "Random variable", "Shape parameter",

386  "Scale Parameter", &cdf\_log);

387

388  // Wrap arguments in vectors

389  VectorView<const T\_y> y\_vec(y);

390  VectorView<const T\_scale\_succ> alpha\_vec(alpha);

391  VectorView<const T\_scale\_fail> beta\_vec(beta);

392  size\_t N = max\_size(y, alpha, beta);

393

394  agrad::OperandsAndPartials<T\_y, T\_scale\_succ, T\_scale\_fail>

395  operands\_and\_partials(y, alpha, beta);

396

397  // Compute CDF and its gradients

398  using boost::math::ibeta;

399  using boost::math::ibeta\_derivative;

400  using boost::math::digamma;

401

402  // Cache a few expensive function calls if alpha or beta is a parameter

403  DoubleVectorView<!is\_constant\_struct<T\_scale\_succ>::value

404  || !is\_constant\_struct<T\_scale\_fail>::value,

405  is\_vector<T\_scale\_succ>::value || is\_vector<T\_scale\_fail>::value>

406  digamma\_alpha\_vec(max\_size(alpha, beta));

407

408  DoubleVectorView<!is\_constant\_struct<T\_scale\_succ>::value

409  || !is\_constant\_struct<T\_scale\_fail>::value,

410  is\_vector<T\_scale\_succ>::value || is\_vector<T\_scale\_fail>::value>

411  digamma\_beta\_vec(max\_size(alpha, beta));

412

413  DoubleVectorView<!is\_constant\_struct<T\_scale\_succ>::value

414  || !is\_constant\_struct<T\_scale\_fail>::value,

415  is\_vector<T\_scale\_succ>::value || is\_vector<T\_scale\_fail>::value>

416  digamma\_sum\_vec(max\_size(alpha, beta));

417

418  DoubleVectorView<!is\_constant\_struct<T\_scale\_succ>::value

419  || !is\_constant\_struct<T\_scale\_fail>::value,

420  is\_vector<T\_scale\_succ>::value || is\_vector<T\_scale\_fail>::value>

421  betafunc\_vec(max\_size(alpha, beta));

422

423  if (!is\_constant\_struct<T\_scale\_succ>::value

424  || !is\_constant\_struct<T\_scale\_fail>::value) {

425

426  for (size\_t i = 0; i < N; i++) {

427

428  const double alpha\_dbl = value\_of(alpha\_vec[i]);

429  const double beta\_dbl = value\_of(beta\_vec[i]);

430

431  digamma\_alpha\_vec[i] = digamma(alpha\_dbl);

432  digamma\_beta\_vec[i] = digamma(beta\_dbl);

433  digamma\_sum\_vec[i] = digamma(alpha\_dbl + beta\_dbl);

434  betafunc\_vec[i] = boost::math::beta(alpha\_dbl, beta\_dbl);

435  }

436  }

437

438  // Compute vectorized CDFLog and gradient

439  for (size\_t n = 0; n < N; n++) {

440

441  // Pull out values

442  const double y\_dbl = value\_of(y\_vec[n]);

443  const double alpha\_dbl = value\_of(alpha\_vec[n]);

444  const double beta\_dbl = value\_of(beta\_vec[n]);

445

446  // Compute

447  const double Pn = ibeta(alpha\_dbl, beta\_dbl, y\_dbl);

448

449  cdf\_log += log(Pn);

450

451  if (!is\_constant\_struct<T\_y>::value)

452  operands\_and\_partials.d\_x1[n] +=

453  ibeta\_derivative(alpha\_dbl, beta\_dbl, y\_dbl) / Pn;

454

455  double g1 = 0;

456  double g2 = 0;

457

458  if (!is\_constant\_struct<T\_scale\_succ>::value

459  || !is\_constant\_struct<T\_scale\_fail>::value) {

460  stan::math::gradRegIncBeta(g1, g2, alpha\_dbl, beta\_dbl, y\_dbl,

461  digamma\_alpha\_vec[n],

462  digamma\_beta\_vec[n], digamma\_sum\_vec[n],

463  betafunc\_vec[n]);

464  }

465  if (!is\_constant\_struct<T\_scale\_succ>::value)

466  operands\_and\_partials.d\_x2[n] += g1 / Pn;

467  if (!is\_constant\_struct<T\_scale\_fail>::value)

468  operands\_and\_partials.d\_x3[n] += g2 / Pn;

469  }

470

471  return operands\_and\_partials.to\_var(cdf\_log);

472  }

473

474  template <typename T\_y, typename T\_scale\_succ, typename T\_scale\_fail>

475  typename return\_type<T\_y,T\_scale\_succ,T\_scale\_fail>::type

476  beta\_ccdf\_log(const T\_y& y, const T\_scale\_succ& alpha,

477  const T\_scale\_fail& beta) {

478

479  // Size checks

480  if ( !( stan::length(y) && stan::length(alpha)

481  && stan::length(beta) ) )

482  return 0.0;

483

484  // Error checks

485  static const char\* function = "stan::prob::beta\_cdf(%1%)";

486

487  using stan::math::check\_positive\_finite;

488  using stan::math::check\_not\_nan;

489  using stan::math::check\_nonnegative;

490  using stan::math::check\_less\_or\_equal;

491  using boost::math::tools::promote\_args;

492  using stan::math::check\_consistent\_sizes;

493  using stan::math::value\_of;

494

495  double ccdf\_log(0.0);

496

497  check\_positive\_finite(function, alpha, "First shape parameter",

498  &ccdf\_log);

499  check\_positive\_finite(function, beta, "Second shape parameter",

500  &ccdf\_log);

501  check\_not\_nan(function, y, "Random variable", &ccdf\_log);

502  check\_nonnegative(function, y, "Random variable", &ccdf\_log);

503  check\_less\_or\_equal(function, y, 1,"Random variable", &ccdf\_log);

504  check\_consistent\_sizes(function, y, alpha, beta,

505  "Random variable", "Shape parameter",

506  "Scale Parameter", &ccdf\_log);

507

508  // Wrap arguments in vectors

509  VectorView<const T\_y> y\_vec(y);

510  VectorView<const T\_scale\_succ> alpha\_vec(alpha);

511  VectorView<const T\_scale\_fail> beta\_vec(beta);

512  size\_t N = max\_size(y, alpha, beta);

513

514  agrad::OperandsAndPartials<T\_y, T\_scale\_succ, T\_scale\_fail>

515  operands\_and\_partials(y, alpha, beta);

516

517  // Compute CDF and its gradients

518  using boost::math::ibeta;

519  using boost::math::ibeta\_derivative;

520  using boost::math::digamma;

521

522  // Cache a few expensive function calls if alpha or beta is a parameter

523  DoubleVectorView<!is\_constant\_struct<T\_scale\_succ>::value

524  || !is\_constant\_struct<T\_scale\_fail>::value,

525  is\_vector<T\_scale\_succ>::value || is\_vector<T\_scale\_fail>::value>

526  digamma\_alpha\_vec(max\_size(alpha, beta));

527  DoubleVectorView<!is\_constant\_struct<T\_scale\_succ>::value

528  || !is\_constant\_struct<T\_scale\_fail>::value,

529  is\_vector<T\_scale\_succ>::value || is\_vector<T\_scale\_fail>::value>

530  digamma\_beta\_vec(max\_size(alpha, beta));

531  DoubleVectorView<!is\_constant\_struct<T\_scale\_succ>::value

532  || !is\_constant\_struct<T\_scale\_fail>::value,

533  is\_vector<T\_scale\_succ>::value || is\_vector<T\_scale\_fail>::value>

534  digamma\_sum\_vec(max\_size(alpha, beta));

535  DoubleVectorView<!is\_constant\_struct<T\_scale\_succ>::value

536  || !is\_constant\_struct<T\_scale\_fail>::value,

537  is\_vector<T\_scale\_succ>::value || is\_vector<T\_scale\_fail>::value>

538  betafunc\_vec(max\_size(alpha, beta));

539

540  if (!is\_constant\_struct<T\_scale\_succ>::value

541  || !is\_constant\_struct<T\_scale\_fail>::value) {

542

543  for (size\_t i = 0; i < N; i++) {

544

545  const double alpha\_dbl = value\_of(alpha\_vec[i]);

546  const double beta\_dbl = value\_of(beta\_vec[i]);

547

548  digamma\_alpha\_vec[i] = digamma(alpha\_dbl);

549  digamma\_beta\_vec[i] = digamma(beta\_dbl);

550  digamma\_sum\_vec[i] = digamma(alpha\_dbl + beta\_dbl);

551  betafunc\_vec[i] = boost::math::beta(alpha\_dbl, beta\_dbl);

552  }

553  }

554

555  // Compute vectorized CDFLog and gradient

556  for (size\_t n = 0; n < N; n++) {

557

558  // Pull out values

559  const double y\_dbl = value\_of(y\_vec[n]);

560  const double alpha\_dbl = value\_of(alpha\_vec[n]);

561  const double beta\_dbl = value\_of(beta\_vec[n]);

562

563  // Compute

564  const double Pn = 1.0 - ibeta(alpha\_dbl, beta\_dbl, y\_dbl);

565

566  ccdf\_log += log(Pn);

567

568  if (!is\_constant\_struct<T\_y>::value)

569  operands\_and\_partials.d\_x1[n] -=

570  ibeta\_derivative(alpha\_dbl, beta\_dbl, y\_dbl) / Pn;

571

572  double g1 = 0;

573  double g2 = 0;

574

575  if (!is\_constant\_struct<T\_scale\_succ>::value

576  || !is\_constant\_struct<T\_scale\_fail>::value) {

577  stan::math::gradRegIncBeta(g1, g2, alpha\_dbl, beta\_dbl, y\_dbl,

578  digamma\_alpha\_vec[n],

579  digamma\_beta\_vec[n], digamma\_sum\_vec[n],

580  betafunc\_vec[n]);

581  }

582  if (!is\_constant\_struct<T\_scale\_succ>::value)

583  operands\_and\_partials.d\_x2[n] -= g1 / Pn;

584  if (!is\_constant\_struct<T\_scale\_fail>::value)

585  operands\_and\_partials.d\_x3[n] -= g2 / Pn;

586  }

587

588  return operands\_and\_partials.to\_var(ccdf\_log);

589  }

590

591  template <class RNG>

592  inline double

593  beta\_rng(const double alpha,

594  const double beta,

595  RNG& rng) {

596  using boost::variate\_generator;

597  using boost::random::gamma\_distribution;

598  // Error checks

599  static const char\* function = "stan::prob::beta\_rng(%1%)";

600

601  using stan::math::check\_positive\_finite;

602

603  check\_positive\_finite(function, alpha, "First shape parameter",

604  (double\*)0);

605  check\_positive\_finite(function, beta, "Second shape parameter",

606  (double\*)0);

607

608  variate\_generator<RNG&, gamma\_distribution<> >

609  rng\_gamma\_alpha(rng, gamma\_distribution<>(alpha, 1.0));

610  variate\_generator<RNG&, gamma\_distribution<> >

611  rng\_gamma\_beta(rng, gamma\_distribution<>(beta, 1.0));

612  double a = rng\_gamma\_alpha();

613  double b = rng\_gamma\_beta();

614  return a / (a + b);

615  }

616

617  }

618 }

619 #endif

stan::agrad::log1m

fvar< T > log1m(const fvar< T > &x)

**Definition:** log1m.hpp:16

stan::prob::beta\_cdf

return\_type< T\_y, T\_scale\_succ, T\_scale\_fail >::type beta\_cdf(const T\_y &y, const T\_scale\_succ &alpha, const T\_scale\_fail &beta)

Calculates the beta cumulative distribution function for the given variate and scale variables...

**Definition:** beta.hpp:213

multiply\_log.hpp

stan::math::gradRegIncBeta

void gradRegIncBeta(double &g1, double &g2, double a, double b, double z, double digammaA, double digammaB, double digammaSum, double betaAB)

**Definition:** internal\_math.hpp:161

traits.hpp

stan::prob::beta\_cdf\_log

return\_type< T\_y, T\_scale\_succ, T\_scale\_fail >::type beta\_cdf\_log(const T\_y &y, const T\_scale\_succ &alpha, const T\_scale\_fail &beta)

**Definition:** beta.hpp:358

stan::agrad::OperandsAndPartials::to\_var

T\_return\_type to\_var(double logp)

**Definition:** partials\_vari.hpp:138

stan::math::check\_positive\_finite

bool check\_positive\_finite(const char \*function, const T\_y &y, const char \*name, T\_result \*result)

**Definition:** check\_positive\_finite.hpp:12

error\_handling.hpp

stan::math::multiply\_log

boost::math::tools::promote\_args< T\_a, T\_b >::type multiply\_log(const T\_a a, const T\_b b)

Calculated the value of the first argument times log of the second argument while behaving properly w...

**Definition:** multiply\_log.hpp:51

stan::length

size\_t length(const T &)

**Definition:** traits.hpp:159

stan::prob::beta\_log

return\_type< T\_y, T\_scale\_succ, T\_scale\_fail >::type beta\_log(const T\_y &y, const T\_scale\_succ &alpha, const T\_scale\_fail &beta)

The log of the beta density for the specified scalar(s) given the specified sample size(s)...

**Definition:** beta.hpp:46

stan::DoubleVectorView

DoubleVectorView allocates double values to be used as intermediate values.

**Definition:** traits.hpp:358

partials\_vari.hpp

stan::agrad::value\_of

T value\_of(const fvar< T > &v)

Return the value of the specified variable.

**Definition:** value\_of.hpp:16

constants.hpp

stan::is\_vector

**Definition:** traits.hpp:83

stan::agrad::lgamma

fvar< T > lgamma(const fvar< T > &x)

**Definition:** lgamma.hpp:15

stan::agrad::OperandsAndPartials

A variable implementation that stores operands and derivatives with respect to the variable...

**Definition:** partials\_vari.hpp:76

traits.hpp

stan::return\_type::type

boost::math::tools::promote\_args< typename scalar\_type< T1 >::type, typename scalar\_type< T2 >::type, typename scalar\_type< T3 >::type, typename scalar\_type< T4 >::type, typename scalar\_type< T5 >::type, typename scalar\_type< T6 >::type >::type type

**Definition:** traits.hpp:406

stan::is\_constant\_struct

Metaprogram to determine if a type has a base scalar type that can be assigned to type double...

**Definition:** traits.hpp:57

stan::math::value\_of

double value\_of(const T x)

Return the value of the specified scalar argument converted to a double value.

**Definition:** value\_of.hpp:24

stan::prob::include\_summand

Template metaprogram to calculate whether a summand needs to be included in a proportional (log) prob...

**Definition:** traits.hpp:35

log1m.hpp

stan::agrad::OperandsAndPartials::d\_x2

VectorView< double \*, is\_vector< T2 >::value, is\_constant\_struct< T2 >::value > d\_x2

**Definition:** partials\_vari.hpp:83

stan::agrad::ibeta

var ibeta(const var &a, const var &b, const var &x)

The normalized incomplete beta function of a, b, and x.

**Definition:** ibeta.hpp:223

stan::math::check\_nonnegative

bool check\_nonnegative(const char \*function, const T\_y &y, const char \*name, T\_result \*result)

**Definition:** check\_nonnegative.hpp:52

stan::math::check\_consistent\_sizes

bool check\_consistent\_sizes(const char \*function, const T1 &x1, const T2 &x2, const char \*name1, const char \*name2, T\_result \*result)

**Definition:** check\_consistent\_sizes.hpp:13

stan::max\_size

size\_t max\_size(const T1 &x1, const T2 &x2)

**Definition:** traits.hpp:191

stan::prob::beta\_rng

double beta\_rng(const double alpha, const double beta, RNG &rng)

**Definition:** beta.hpp:593

internal\_math.hpp

stan::math::check\_less\_or\_equal

bool check\_less\_or\_equal(const char \*function, const T\_y &y, const T\_high &high, const char \*name, T\_result \*result)

**Definition:** check\_less\_or\_equal.hpp:52

stan::math::check\_not\_nan

bool check\_not\_nan(const char \*function, const T\_y &y, const char \*name, T\_result \*result)

Checks if the variable y is nan.

**Definition:** check\_not\_nan.hpp:56

stan::agrad::digamma

fvar< T > digamma(const fvar< T > &x)

**Definition:** digamma.hpp:16

value\_of.hpp

stan::prob::beta\_ccdf\_log

return\_type< T\_y, T\_scale\_succ, T\_scale\_fail >::type beta\_ccdf\_log(const T\_y &y, const T\_scale\_succ &alpha, const T\_scale\_fail &beta)

**Definition:** beta.hpp:476

stan::agrad::OperandsAndPartials::d\_x1

VectorView< double \*, is\_vector< T1 >::value, is\_constant\_struct< T1 >::value > d\_x1

**Definition:** partials\_vari.hpp:82

stan::agrad::OperandsAndPartials::d\_x3

VectorView< double \*, is\_vector< T3 >::value, is\_constant\_struct< T3 >::value > d\_x3

**Definition:** partials\_vari.hpp:84

stan::agrad::log

fvar< T > log(const fvar< T > &x)

**Definition:** log.hpp:15

stan::VectorView

VectorView is a template metaprogram that takes its argument and allows it to be used like a vector...

**Definition:** traits.hpp:275

stan::math::log1m

boost::math::tools::promote\_args< T >::type log1m(T x)

Return the natural logarithm of one minus the specified value.

**Definition:** log1m.hpp:40


---

[ Stan Home Page ]

*© 2011–2014,
Stan Development Team.*
